# Advances in the cell biology of the trafficking and processing of amyloid precursor protein: impact of familial Alzheimer's disease mutations

**DOI:** 10.1042/BCJ20240056

**Published:** 2024-09-20

**Authors:** Jingqi Wang, Lou Fourriere, Paul A. Gleeson

**Affiliations:** Department of Biochemistry and Pharmacology, Bio21 Molecular Science and Biotechnology Institute, The University of Melbourne, Melbourne, Victoria 3010, Australia

**Keywords:** Alzheimers disease, amyloid precursor protein, endosomal sorting, Golgi apparatus, neurons, trafficking

## Abstract

The production of neurotoxic amyloid-β peptides (Aβ) is central to the initiation and progression of Alzheimer's disease (AD) and involves sequential cleavage of the amyloid precursor protein (APP) by β- and γ-secretases. APP and the secretases are transmembrane proteins and their co-localisation in the same membrane-bound sub-compartment is necessary for APP cleavage. The intracellular trafficking of APP and the β-secretase, BACE1, is critical in regulating APP processing and Aβ production and has been studied in several cellular systems. Here, we summarise the intracellular distribution and transport of APP and its secretases, and the intracellular location for APP cleavage in non-polarised cells and neuronal models. In addition, we review recent advances on the potential impact of familial AD mutations on APP trafficking and processing. This is critical information in understanding the molecular mechanisms of AD progression and in supporting the development of novel strategies for clinical treatment.

## Introduction

Alzheimer's disease (AD), characterised by dementia, cognitive and behavioural disabilities most commonly amongst the aged, is one of the most prevalent neurodegenerative disorders affecting more than 46.8 million people worldwide [1,2]. There is currently no cure for the disease, and the exact mechanism(s) of disease progression remains poorly defined (reviewed in [3–5]). To date, the accumulation of amyloid-β plaques and tangles in the brain are the most well-established hallmarks of AD [1]. During the past three decades, researchers have strived to understand, as well as to prevent, the formation of the extracellular amyloid-β aggregates and amyloid plaques. Neurotoxic amyloid-β peptides (Aβ) are generated within the cell, and most of the molecular players along the amyloid production pathway are membrane-bound proteins [5,6]. Dysregulated trafficking of these membrane proteins in the cell can result in aberrant co-residency of substrate and proteolytic enzyme and, subsequently, the emergence of toxic protein products prone to aggregation. Hence, it is vital to investigate the intracellular trafficking and processing sites of the key proteins involved in the generation and secretion of toxic Aβ, and the subsequent amyloid deposits, to understand the pathogenesis of AD.

The process of protein trafficking is critical in maintaining cellular homeostasis. Defects in protein sorting and membrane trafficking are tightly linked with the development of a range of neurological diseases, including AD, Parkinson's disease, Huntington's disease, Down syndrome, amyotrophic lateral sclerosis and prion disorders (reviewed in [7,8]). Both the secretory and endocytic membrane trafficking pathways have been implicated in the progression of these diseases: dysregulated Golgi sorting and the perturbation of Golgi morphology can contribute to the neuropathology [9,10] and defects in the endocytic system as well as lysosomal degradation can also lead to the emergence of neurological diseases [11,12].

Here, the intracellular trafficking and location of key membrane proteins involved in APP processing are reviewed in non-specialised cells as well as in neuronal models, information relevant to identifying the molecular pathways contributing to the production of toxic Aβ and the initiation and progression of AD. Understanding how dysregulation of membrane trafficking promotes neurodegeneration is relevant to design novel approaches to inhibit and/or reduce the progression of AD.

## AD and the amyloid cascade model

AD has the highest economic and health costs of the neurodegenerative diseases, with no effective treatment available. The aetiology and pathogenesis of the disease remain uncertain. Although autosomal dominant mutations have been identified in familial, early onset, AD, more than 95% of the cases are sporadic with late-onset where symptoms occur at or after age 65, and involve both genetic and environmental risk factors [1]. Hallmarks of AD include amyloid accumulation in the brain (also called amyloid-β or Aβ plaques), neurofibrillary tangles which consist of Tau aggregates, and chronic inflammation in the brain observed in Alzheimer's patient brain and, with some features shared in animal models (reviewed in [4,13,14]). Abnormal intracellular production of Aβ has been intensively studied, as many familial disease-related mutations identified in early onset disease are associated with the amyloid production pathway, namely mutations associated with amyloid precursor protein (APP) and its cleaving enzyme, the PSEN1/PSEN2 γ-secretase [15]. Genetic risk factors have been identified associated with late onset sporadic cases, with the apolipoprotein gene *APOE4* being the most significant influencing risk factor [16]. Other risk alleles of AD are associated with dysfunctions in vesicular trafficking, cholesterol metabolism and innate immune responses [4,16].

The sequence of events in the initiation and progression of AD is controversial; however, the cell processes associated with inheritable familial disease and sporadic late onset cases collectively involve the production of amyloid peptides and membrane trafficking pathways [16]. Of note is the identification of a protective APP mutation for AD [17], which is associated with reduced APP amyloidogenic processing and Aβ production, providing further evidence that the generation of Aβ seeds the onset of the disease. Furthermore, there is also a recognition that the recruitment of additional pathological processes, mediated by abnormal Aβ levels, namely inflammation and tauopathy, play important roles in contributing to the progression of the disease [4,13].

The amyloid cascade model, proposed by Hardy and Higgins [6], speculated that the production of Aβ and the accumulation of Aβ aggregates and plaques were responsible for the neurodegeneration in AD. Aβ peptides are generated through proteolytic processing of the APP. APP, first identified by Kang et al. [18], is a type I transmembrane protein with a long luminal N-terminal domain and a small cytoplasmic C-terminal tail [18,19]. There are three major isoforms of APP, derived from alternative splicing, which differ in length [20]. The APP_695_ isoform is predominantly expressed in the brain [21] and relevant for the generation of Aβ peptides.

**Figure 1.**
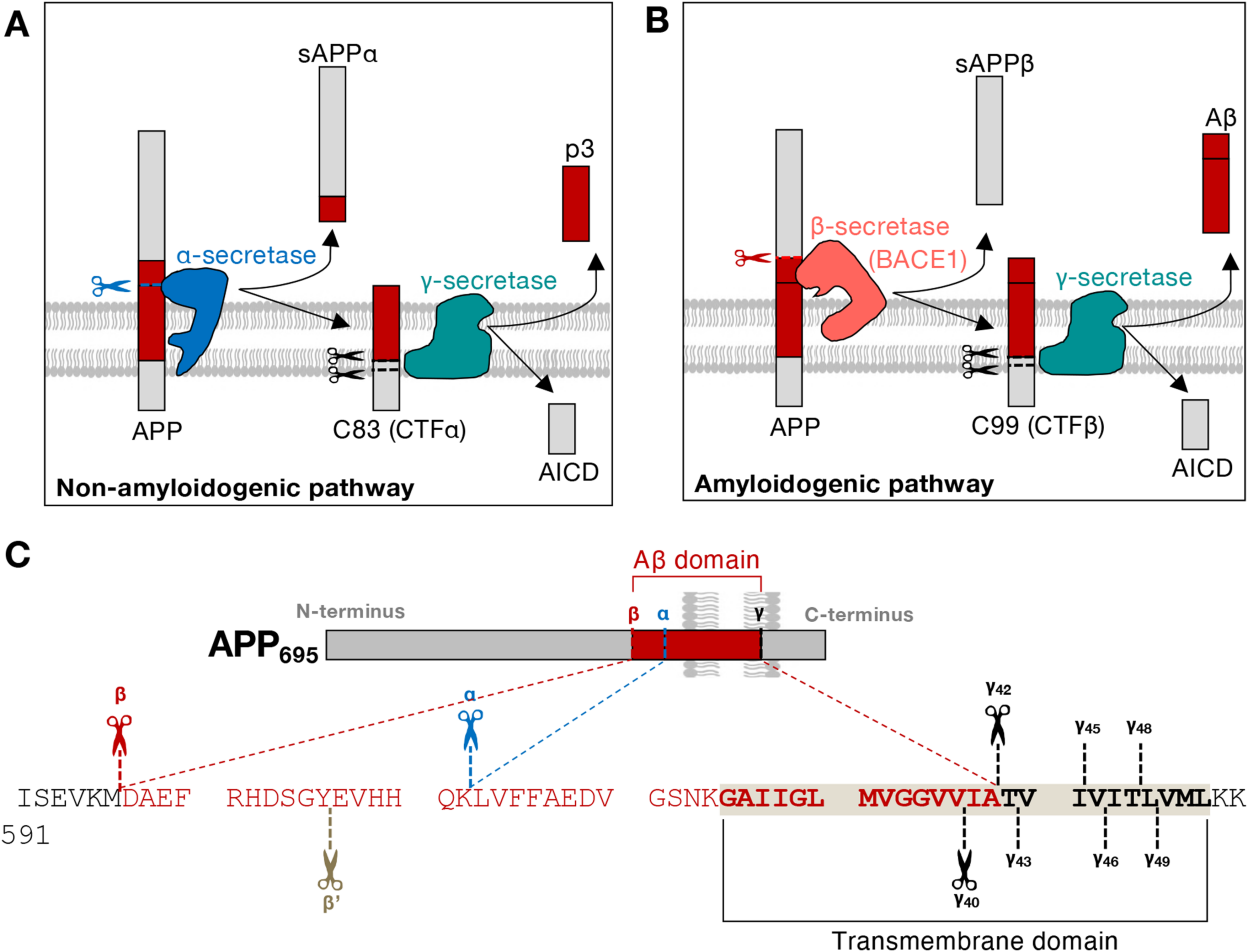
APP processing. Full-length APP can be cleaved sequentially by different secretases to give rise to different products. (**A**) In the non-amyloidogenic pathway, APP is cleaved by α-secretase, producing sAPPα and C83 (CTFα) fragments. C83 (CTFα) can be further cleaved by γ-secretase, generating p3 and AICD fragments. p3 is not neurotoxic. (**B**) In the amyloidogenic pathway, APP is cleaved by β-secretase, producing sAPPβ and C99 (CTFβ). Subsequent cleavage of C99 (CTFβ) by γ-secretase leads to the formation of AICD and Aβ. Soluble Aβ can be secreted and aggregate into amyloid plaques, a hallmark of Alzheimer's disease. (**C**) The protein sequence of APP_695_ close to the secretase cleavage sites. Sequence of the transmembrane domain (TMD, in **bold**), and flanking sequences of the cytoplasmic tail and the luminal domain of APP are shown. The cleavage sites of α-, β- and γ-secretases are illustrated in blue, red and black, respectively.

APP undergoes a series of cleavage events by different enzymes known as the secretases (reviewed in [19]) (Figure 1). In the non-amyloidogenic pathway, APP is first cleaved by the α-secretase, generating the soluble and membrane-bound fragments, sAPPα and C83, respectively [22,23] (Figure 1A). In contrast, in the amyloidogenic pathway, APP is cleaved by the β-secretase (BACE1), producing sAPPβ and C99 [24] (Figure 1B). Both C83 and C99, which are the APP C-terminal fragments CTFα and CTFβ, respectively, are subsequently cleaved by the γ-secretase [25,26]. Two distinct products, p3 or Aβ, are generated from C83 and C99, alongside the liberated APP intracellular domain (AICD) [25,26] (Figure 1). Secreted Aβ can oligomerise, induce inflammation and aggregate into potentially toxic amyloid plaques [27,28], while p3 is not known to be neurotoxic. Nanomolar levels of soluble Aβ oligomers are known to be highly cytotoxic and mediate damage via a number of cellular pathways, including the formation of Aβ membrane channels [29,30]. Together with the full length APP, fragments of APP after secretase processing are implicated in neuronal maintenance and synaptic function [31]. Soluble N-terminal APP fragments can act as trophic factors [32,33] and AICD can mediate transcriptional activation, by an indirect mechanism involving membrane-associated AICD acting as a scaffold to recruit and activate the nuclear adaptor protein, Fe65 [34–36]. In addition to signalling, a recent study has shown that elevated levels of the APP CTFs, localised to membrane contacts sites between late endosomes/lysosomes and the endoplasmic reticulum (ER), drives the dysregulation of lysosomal function [37].

Since APP and the secretases are transmembrane proteins, they must first co-localise in the same membrane compartment, and in the same membrane subdomain, for the cleavage event to take place. Hence, the trafficking and distribution of APP and its secretases must be tightly regulated to control APP processing and Aβ production and dysregulation of APP intracellular trafficking may be a primary event associated with AD.

### Main challenges to define the intracellular locations of APP processing

The intracellular distribution and itinerary of three membrane proteins, namely APP, BACE1 and γ-secretase need to be defined to identify potential compartments for APP processing and Aβ production. Defining these pathways and processes that are relevant to primary neurons has been very challenging [38]. To appreciate the technical issues and their impact on the interpretation of the literature in this field over the past 30+ years, the following issues need to be considered.

1. A range of immortalised cell lines have been used to define the membrane transport of the three membrane cargoes and the location of processing events. A range of different cell lines have been employed and the comparison of data between cell types is not straightforward to reconcile. In some studies, primary neurons have been included to validate findings.
2. The potential impact of overexpression on APP trafficking and processing needs to be considered in studies which have relied exclusively on overexpression.
3. The interpretation of experiments involving mutation of sorting motifs of APP and BACE1, as well as involving adaptor proteins, can be problematic when the sorting motif or adaptor can act at more than one location in the cell, as is the case in some instances.
4. The specificity of the antibodies used to detect intracellular APP needs to be rigorously defined as they often have the potential to recognise several APP fragments.
5. The development of super-resolution optical microscopy has dramatically improved the quality of data and the ability to define the precise co-localisation of APP and secretases (∼100 nm resolution), whereas early studies (1990s and 2000s) to measure the degree of overlap of APP and secretases were limited by the resolution available at that time. For example, super-resolution microscopy has revealed the subcellular localisations of the APP products in primary neurons [39] and low level of co-localisation of APP and BACE1 early in the secretory pathway [40].
6. Quantitative analysis of optical and electron microscopy (EM) imaging is essential for defining the distribution of APP, secretases and products in different cellular compartments and is often lacking.
7. The intracellular location of Aβ is usually assessed under steady state conditions, and may not reflect the source where Aβ is generated. The Aβ peptide is likely to be partitioned between soluble luminal contents and luminal membranes; once produced Aβ could traffic to other locations via different transport pathways along the endocytic pathway, from the endocytic pathway to the Golgi, from the Golgi to the early endosomes/late endosomes and by retrograde transport from the early endosomes and late endosomes to the Golgi and to the ER [41].
8. Some studies have incorporated multiple familial mutants in the same line or mouse transgenic, and the impact of the individual independent mutations on trafficking and processing of the cargo can be difficult to assess and relate to physiological conditions.

Finally, it is clear that there are multiple intracellular compartments where APP processing can occur and the relative contribution of these compartments can differ between healthy conditions and the pathological conditions. The impact of the experimental protocol and read-outs need to be considered in identifying the dominant processing compartment for familial mutations and risk alleles.

In this review, we will summarise the current evidence on (a) the intracellular distribution and trafficking of APP, (b) the intracellular processing locations of APP and (c) the effect of selected familial AD mutations on APP intracellular trafficking and processing and the production of Aβ, taking the above challenges into account.

## Intracellular distribution of APP

### Trafficking and sorting of APP in non-neuronal cells

As APP and the secretases are all membrane-bound proteins, the co-localisation of APP and its secretases is required for cleavage events to occur. Thus, the sorting and transport of APP and the secretases in the cell are critical for the regulation of APP processing. Below is a summary of APP trafficking and sorting, complimented by a number of previous reviews [42–46].

**Figure 2.**
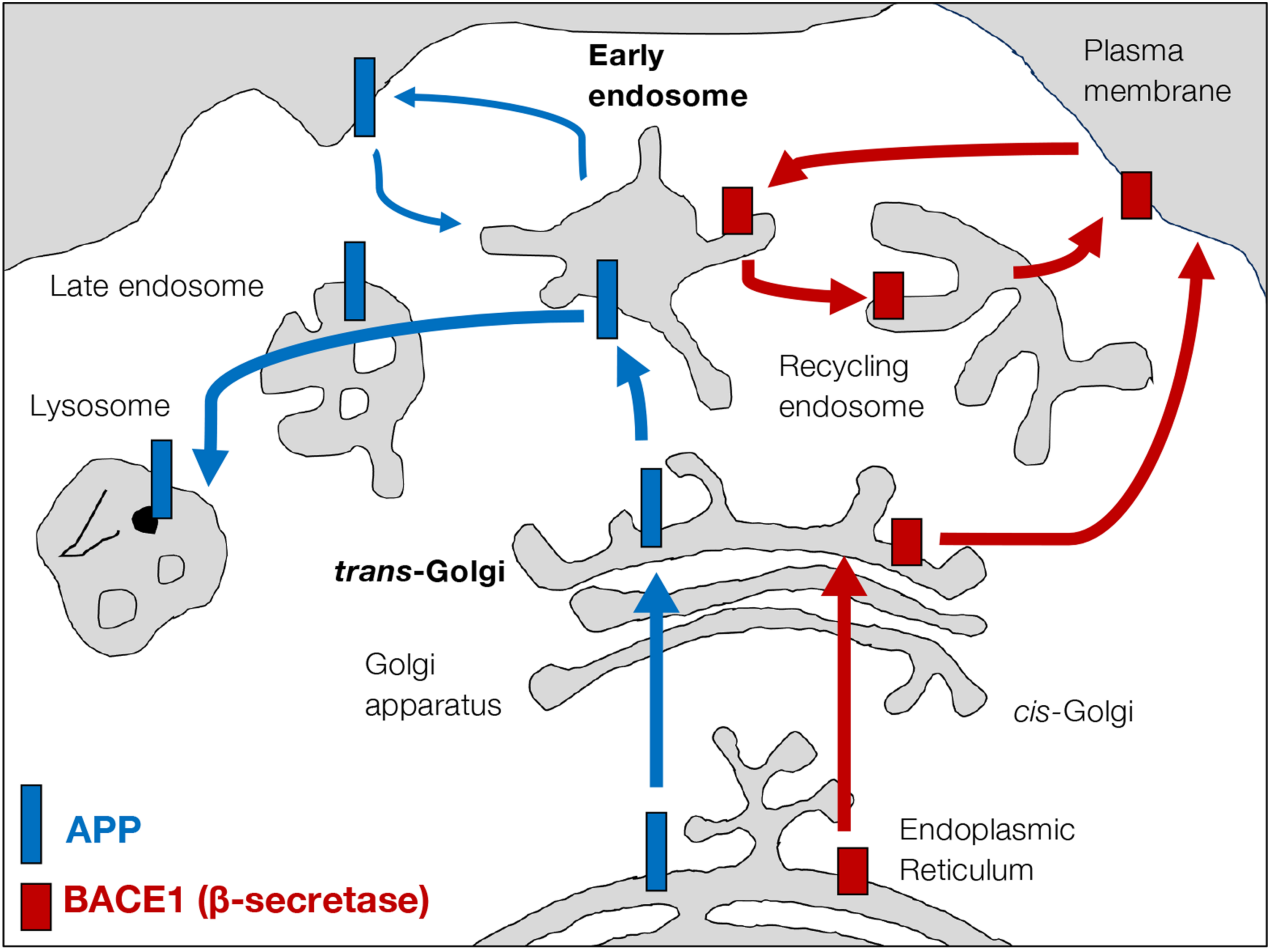
Trafficking itineraries of APP and the β-secretase BACE1 in non-polarised cells. Intracellular trafficking itineraries of APP and the β-secretase BACE1 in non-polarised cells are illustrated in blue and red, respectively. Both APP and BACE1 are synthesised in the endoplasmic reticulum (ER) and transported to the Golgi apparatus. APP and BACE1 segregate within the Golgi stack and are sorted into different transport carriers at the *trans*-Golgi network (TGN). The bulk of APP is transported to the early endosomes and then transported along the late endosome-lysosome pathway in cultured cells using protocols to synchronise trafficking of APP from the ER [47,48]. Low levels of APP are transported to the cell surface as quantified by TIRF [48] and are present at steady state as assessed by subcellular fractionation [49]. In contrast, BACE1 is sorted directly to the plasma membrane [50]. BACE1 can be actively internalised and recycled through the recycling endosomes, while APP showed minimal localisation to the recycling endosomes. Hence, the TGN and the early endosomes serve as potential sites for the β-cleavage of APP.

APP trafficking and intracellular distribution was first described in non-neuronal cells (reviewed in [44,45]) (Figure 2). As for most transmembrane proteins, full-length APP is synthesised in the ER and transported to the Golgi apparatus for post-translational modifications and sorted into a post-Golgi transport pathway [51,52]. Conventionally, APP was considered to be transported to the cell surface (plasma membrane, PM) from the *trans*-Golgi network (TGN) [42,46], and then endocytosed to the early endosomes through clathrin-AP2 mediated endocytosis [53–56]. In more recent studies, APP was observed to be transported directly from the TGN to the endosomes, a pathway dependent on the adaptor protein AP4 [57] and Arl5b [47]. From the early endosomes, the majority of APP is then degraded along the late endosomal-lysosomal pathway [56,58] and with only low levels reaching the PM as quantified by TIRF and inhibition of endocytosis [47,48] (Figure 2). In addition, APP cargo has been observed to traffic back to the TGN from the early endosomes through the retromer-mediated retrograde transport [59].

Several sorting motifs in the cytosolic domain of APP have been identified. The tyrosine-based YKFFE motif can be recognised by the adaptor protein AP-4 at the TGN [47,57]. Mutation of the tyrosine or phenylalanine residues in YKFFE, or AP-4 depletion to disrupt the interaction between APP and AP-4, resulted in accumulation of APP in the TGN [47,57]. Another tyrosine-based motif in the cytoplasmic tail of APP, YENPTY, can bind to various scaffold proteins such as MINT [60–63] and Shc adaptor proteins [64,65], which mediate the endosomal/lysosomal transport of APP (reviewed in [66]). In addition, the tyrosine-based YTSI motif in the cytoplasmic tail can interact with the adaptor protein AP-3, to mediate APP traffic to the lysosomes for degradation [67]. Collectively, these motifs and interacting partners divert APP from a TGN-PM pathway to a TGN-endo-lysosomal pathway in non-neuronal cells.

APP has eight potential phosphorylation sites in the cytoplasmic domain and seven of these phosphorylation sites are found in brains of patients with AD 2003 [68]. Some of these phosphorylation sites regulate APP trafficking and processing. For example, phosphorylation of S655 of the YTSI endocytosis motif regulates the endosomal trafficking to the late endosomes/lysosomes and the TGN [69] . For extensive details on the role of phosphorylation of APP sorting motifs and APP trafficking and processing see reviews by [45,70].

### Polarised trafficking and processing of APP in primary neurons

A critical aspect in the neuronal production of Aβ is the complexity of APP trafficking and processing in the highly polymorphic neuronal cell. Neurons are polarised cells with extensions from the cell body, or soma, called axons and dendrites. Understanding the cell biology of APP trafficking and processing in primary neurons has been a challenge due to the size and shape of neurons, and the technical challenge in maintaining these differentiated cells in culture. As a consequence, there remain considerable gaps in identifying the pathways and sites of APP processing in primary neurons. In this section, we review the current knowledge of the APP localisation in neuronal cells.

### Intracellular distribution of APP in the polarised domains of rodent neurons

In rodent brains, APP is expressed predominantly by neurons, rather than astrocytes, glial cells or microglia [71]. The intracellular distribution of APP in neurons has been predominantly studied using immunoelectron microscopy (EM) or immunofluorescence (IF) of rodent brain sections or primary rodent neuronal cultures, using antibodies targeting different domains of endogenous APP (either N- or C-terminal domains) in combination with various organelle markers as summarised in Table 1.

**Table 1.** Intracellular localisation of the endogenous APP in different primary neuron models

Location	APactP || organelle marker	Methods	Neuronal cell type	References
ER and ERGIC	APP (C-ter) **||** KDEL	IF	Primary mouse hippocampal neuron	[71]
Golgi/TGN	APP (N- and C-ter) **||** Golgi *cisternae* membranes	EM	Neuron in rat brain section	[72,73]
	APP (C-ter) **||** GIMP	IF		[74]
	APP (C-ter) **||** Golgi *cisternae* membranes	EM		
	APP (C-ter) **||** GM130	IF	Primary mouse hippocampal neuron	[71]
Plasma membrane (PM)	APP	IF	Neuron in rat brain section	[75]
	APP (N-ter)	EM		[73]
Early endosomes	APP (C-ter) **||** clathrin heavy chain	IF	Primary rat hippocampal neuron	[76]
	APP (C-ter) **||** EEA1	IF	Primary mouse hippocampal neuron	[71]
	APP **||** Vps35/EEA1	IF		[77]
Late endosomes	APP (C-ter) **||** M6PR	IF		[71]
	APP (C-ter) and C83/C99 (CTFs)	isolated exosomes	Primary rat cortical neurons	[78]

The distribution of endogenous APP fragments in various rodent neuronal models. The localisation of endogenous APP was detected using antibodies targeting either N-terminal or C-terminal domains of APP together with organelle markers, by electron microscopy (EM) or immunofluorescence (IF).

Many studies have investigated the organellar location of APP in the cell body. Consistent with the findings in non-polarised cells, somatic APP resides predominantly in the Golgi and early or late endosomes in neurons (Table 1) and very little APP is detected in the lysosomes [71]. The trafficking dynamics of APP from the cell body to the neuronal extensions and synapses is important in understanding the function of APP in neuronal cells and its role in the development of AD.

**Figure 3.**
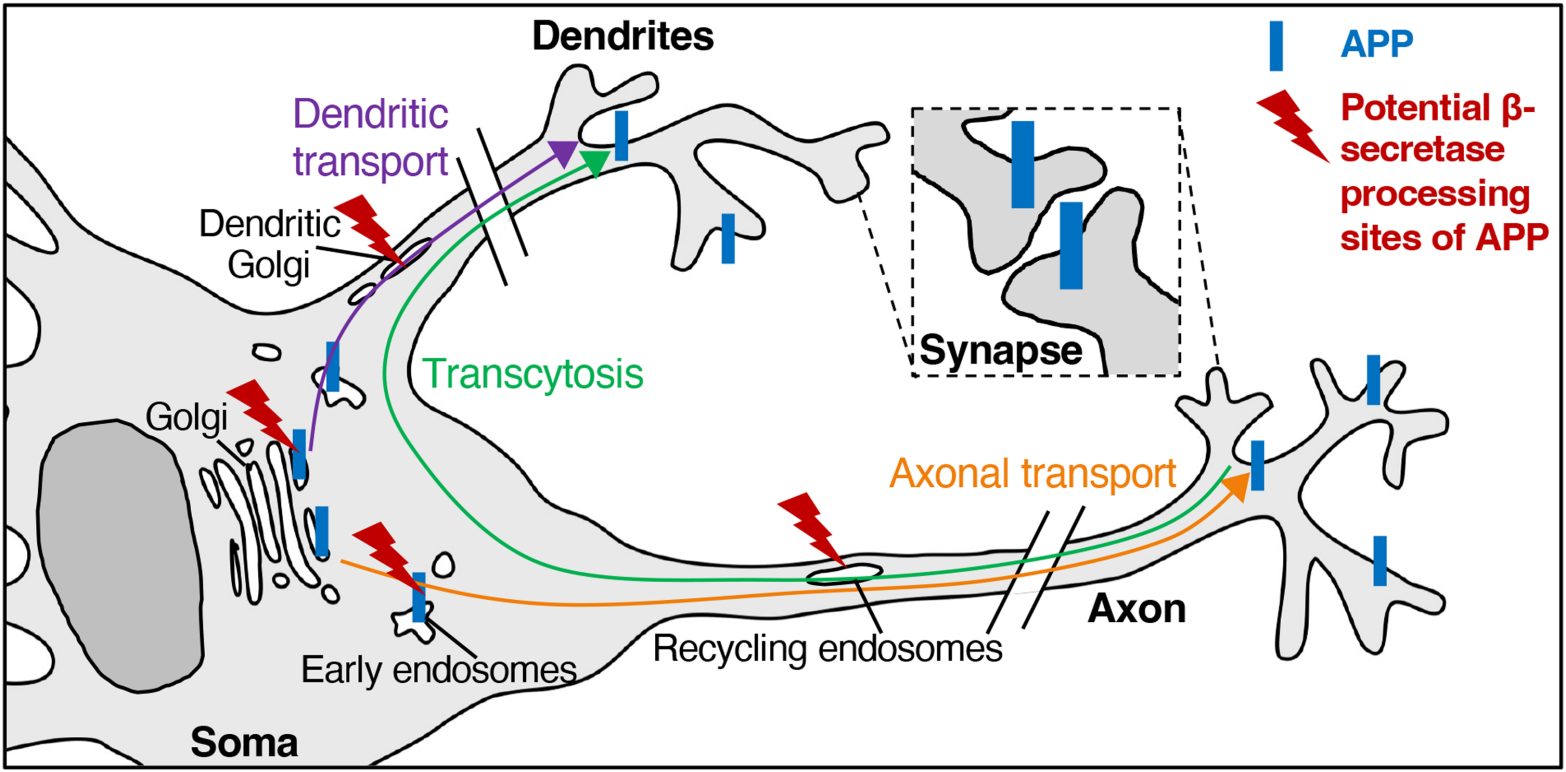
APP trafficking and sites of processing in primary rodent neurons. APP trafficking in primary rodent neuronal models is complex, as neurons are comprised of polarised subdomains (axon and dendrites) in addition to the cell body (soma). APP is distributed in primary rodent neurons in the soma, dendrite, and axon, as well as both pre- and post-synapses. In the soma, APP is mainly associated with the Golgi and Golgi-derived vesicles, and the early and recycling endosomes. Newly synthesised APP can be transported to the axon and dendrites. APP can also be transcytosed from the axonal domain to the dendrites. Limited levels of APP and BACE1 co-localisation have been observed in the Golgi and Golgi-derived vesicles, as well as recycling endosomes.

Most studies have reported that APP is enriched in the perinuclear region in the soma, as well as punctate structures widely distributed in neurites (Figure 3). In primary rodent neurons APP localised to protruding neuronal processes visualised by IF [74,76,79], and APP puncta (C-terminus) were detected in both MAP2-positive dendrites and MAP2-negative axons [76]. These observations were confirmed by EM where APP was also observed in myelinated axons and unmyelinated nerve fibres [73,74]. Furthermore, exogenously expressed human APP_695_ has been detected on both axonal and dendritic surfaces in primary rodent neurons by several laboratories [80–82]. In addition, endogenous APP was detected in vesicles closely associated with the pre- and post-synaptic membranes [73,74] (Figure 3) including synaptic vesicles in rat brain [83].

An important finding is that, once APP is cleaved, the N- and C-terminal fragments (CTFs/NTFs) of APP are observed in distinct compartments or vesicles in neurons, as demonstrated using specific antibodies targeting either the N- or the C-terminal sequences of APP [39,79] and live cell imaging using N- and C-termini-tagged APP constructs [84]. APP CTFs were found predominantly in punctate structures concentrated in the soma, while NTFs were located in membrane structures in neurites associated with short cytoskeletal filaments [84]. This observation most likely reflects differential transport pathways for full length APP and APP cleaved products, and emphasises their different neuronal distribution, and the need to use a combination of antibodies targeting different domains of APP, to identify APP fragments by IF.

### Dynamic dendritic and axonal transport of tagged-APP in live neurons

The transport itinerary of APP in neurons has been investigated using tagged-APP in live primary rodent neurons. Axonal transport and transcytosis have been demonstrated for APP trafficking (Figure 3). Exogenous expression of c-myc-tagged APP_695_ in rat hippocampal neurons was detected initially in the cell body and neurofilament-labelled axons 3 h after its expression, and then in the MAP2-positive dendrites 5 h after its expression [80]. This observation suggests that APP is initially transported to the axon before being transcytosed to the dendrites. Indeed, a number of subsequent studies have confirmed a preferential axonal transport of APP in primary rodent neurons: the axonal transport of YFP-tagged APP was observed in fast, tubular carriers in live rat hippocampal neurons [85]. In addition, the axonal trafficking of APP-mCherry was impaired by the expression of toxic α-synuclein in primary mouse neurons [86]. Furthermore, the axonal APP transport has been shown to be regulated by the phosphoinositide 3-kinase (PI3K), PI3Kδ; expression of the dominant-negative inactive PI3Kδ induced the formation of tubular APP carriers with bi-directional movement and reduced APP transport in the axon [87]. The microtubular motors, kinesin and dynein, play a role in regulating the axonal trafficking of APP. A number of microtubule motor regulatory factors have been identified as potential regulators of APP axonal transport, such as JIP1 [88,89], Rab3A [90], Calsyntenin-1 [91] and phosphorylated kinesin light chain-1 [92] in primary rodent neurons.

The APP sequences regulating APP polarised neuronal trafficking remain poorly defined. APP is transported to both pre- and post-synaptic sites. Deletions of either APP N- or C-terminal sorting motifs did not affect APP delivery to the axonal or dendritic domains in primary mouse cortical neurons [81]. Mutation of the APP YENPTY motif, which is responsible for APP endocytosis in non-polarised cells, did not alter APP endosomal distribution in H9 human embryonic stem cell-derived neurons [93]. These findings suggest that polarised APP trafficking in the axon and dendrites is independent of the well-defined sorting sequences of APP identified in non-polarised cells. Therefore, further analysis is required to identify the relevant signals for APP polarised transport in primary neurons.

Missorted APP to either the axons or the dendrites has been shown to be associated with altered APP secretase processing. In primary rat hippocampal neurons, a chimeric APP with a LDLR cytoplasmic tail was artificially targeted to dendrites, which resulted in an increase in Aβ production compared with a chimeric APP with a NgCAM cytoplasmic sequence artificially targeted to the axons [82]. On the other hand, increased β-secretase processing of APP was shown to reduce APP anterograde axonal transport, and inhibition of β-cleavage can stimulate APP anterograde axonal transport in live mouse hippocampal neurons [94]. Hence, alterations in APP transport to the axonal or somatodendritic domain is associated with a modification of APP processing.

## Intracellular location of APP processing

### Location of APP processing by β-secretase (BACE1)

β-cleavage of APP (by BACE1) is the rate-limiting step of the amyloidogenic pathway. Therefore, the trafficking and localisation of BACE1, and the convergence of APP and BACE1 at intracellular sites is critical to understand the regulation of APP processing (reviewed in [95,96]).

### Convergence of APP and BACE1 in non-polarised cells

BACE1 is synthesised in the ER and transported to the Golgi apparatus along the secretory pathway. At the TGN, the bulk of BACE1 is delivered directly to the PM and endocytosed back to the early endosomes by AP2-clathrin mediated endocytosis [97,98] (Figure 2). BACE1 recycles back to the PM via the Rab11 recycling endosomes [97,99] (Figure 2). The DDISLL motif on the cytoplasmic tail of BACE1 can interact with a range of adaptor proteins, including AP1 for anterograde TGN sorting, AP2 for endocytosis [97,98], GGA family proteins for retrograde transport back to the TGN [100,101] and to the recycling endosomes [99].

Phosphorylation of BACE1 also regulates its intracellular trafficking [43], notably phosphorylated S498 in the BACE1 DDISL motif enhances the binding of BACE1 by GGA [101], which regulates trafficking of BACE1 from the early endosomes to the recycling endosomes in cell lines and primary neurons [99]. Other studies have reported that the phosphorylation of S498 enhances retrograde transport from the early endosomes to the TGN [101–103]. The non-phosphorylated S498A BACE1 was shown to have prolonged residency in the early endosomes and enhanced Aβ production compared with the phosphomimic S498D BACE1 [99].

While APP is predominately located to the Golgi and the endosome-lysosome pathway, the majority of BACE1 resides in the Golgi and in the recycling pathway between the recycling endosome and the PM (Figure 2). Hence, two locations are identified as prime sites for APP and BACE1 convergence: [1] the Golgi, especially the TGN, and [2] the early endosomes (Figure 2). Importantly, the TGN [104] and early endosomes [105] provide an acidic environment which is optimal for BACE1 catalytic activity [106].

The level of APP processing is highly regulated by several mechanisms to segregate APP and BACE1 throughout the secretory pathway. Super-resolution microscopy has recently identified a strong segregation between APP and BACE1 on exit from the ER and throughout the Golgi apparatus. This segregation is likely to reflect the localisation into different subdomains of the membranes of the Golgi *cisternae* [40]. APP and BACE1 interact with different adaptor complexes at the TGN are sorted into distinct post-TGN trafficking pathways: TGN exit of APP is dependent on AP4 and Arl5b, while BACE1 is affected by AP1 and Arf1/4 [47,50]. Hence, the segregation of BACE1 and APP throughout the secretory pathway may provide an important mechanism to protect APP from excessive β-secretase cleavage under normal physiological conditions and alterations in transport kinetics or localisation of either APP or BACE1 can dramatically change Aβ production.

### Intracellular colocalisation of APP and BACE1 in primary neurons

In healthy neurons, only a low level of co-localisation of APP and BACE1 has been observed. Exogenous APP-GFP and BACE1-mCherry in primary mouse hippocampal neurons were localised in distinct carriers: BACE1-mCherry localised in transferrin receptor (TfR)-positive recycling endosomes, while APP-GFP localised to neuropeptide-Y signal-sequence (NPYss)-marked Golgi-derived vesicles [107]. In resting, non-stimulated conditions, APP-GFP and BACE1-mCherry did not co-localise in rat primary hippocampal neurons [108] or chick retinal ganglion cells [109]. Moreover, from crude mouse brain, immuno-isolated APP-containing membrane fractions did not contain BACE1 (β-secretase) or PSEN1 (γ-secretase), however, ADAM10 (α-secretase) was present [90]. Collectively, these results indicate that the majority of APP and BACE1 do not reside in the same compartment in neurons.

Under physiological conditions there is only a limited convergence of APP and BACE1 in neurons; nonetheless it is still important to identify where APP and BACE1 co-localise as it influences APP processing. A low level of co-localisation between endogenous APP and BACE1-GFP was observed in axons of primary mouse hippocampal neurons [110]. A bimolecular fluorescence complementation method has been employed in primary mouse hippocampal neurons [111]. APP was tagged with the N-terminal domain of the fluorescent protein Venus (VN), and BACE1 with the C-terminal domain of Venus (VC). APP-VN and BACE1-VC interactions were observed in: (1) galactosyl transferase (GalT)-positive Golgi in the soma, (2) TfR- or Rab11-positive recycling compartments in dendrites and (3) NPYss-marked Golgi-derived vesicles in the axons [111]. There is evidence that the extent of convergence of APP and BACE1 in neurons regulates Aβ production. For example, by exploiting a US9-derived protein gPTB9TM to direct APP away from BACE1 in neurons, β-secretase processing of APP is reduced without affecting physiological BACE1 activities [112].

The effect of synaptic activity on APP-BACE1 convergence has been investigated. In primary mouse hippocampal neurons, glycine-induced stimulation of primary mouse hippocampal neurons increased the co-localisation of APP-GFP and BACE1-mCherry [107]. Neuronal stimulation re-directed APP-GFP into the recycling endosomes and the subsequent increase in β-secretase cleavage products of APP, namely C99 (CTFβ) [107]. Neuronal activity has also been linked with increased Aβ secretion. In mouse hippocampal sections expressing a pathogenic familial APP mutation, APP Swedish, stimulation of neuronal activity led to an increase of Aβ secretion as well as an elevation of C99 (CTF-β) levels detected [113]. Induced electrical stimulation of neurons also resulted in increased Aβ in the interstitial fluid of mouse brains *in vivo* [114]. Altogether, these findings suggest that synaptic activity could enhance the co-residence of BACE1 and APP, resulting in an increase in APP processing and Aβ production in neurons, by modulating APP trafficking.

### Detection of products of intracellular β-secretase processing of APP

Many studies have investigated the intracellular sites of β-secretase cleavage of APP, often by detecting the BACE1 cleavage products of APP, namely sAPPβ, C99 (CTFβ) and Aβ (Figure 1). β-Secretase processing products of APP have been found in both the early secretory pathway and the endocytic system. Findings from these different studies are summarised in Table 2 and are discussed in the subsequent sections.

**Table 2.** Detection of β-secretase cleavage products of APP in different cellular compartments

Location	References	Cleavage products detected and methods	Cell type	APP expression
ER and ERGIC	[115]	Aβ_42_ detected in the ER by electron microscopy using a specific antibody.	Primary rat hippocampal neurons	Endogenous and overexpression
	[116]	Aβ_42_ co-localised with an ER marker by IF.	Primary rat cortical neurons	Endogenous
		Aβ_42_ detected in the ER fraction by subcellular fractionation.	N2a Mouse Neuroblastoma	Overexpression
	[117]	C99 (CTFβ) was increased in the ER fraction by subcellular fractionation in PSEN1 KO.	Fibroblasts from KO mouse embryo	Endogenous
		Aβ_42_ detected in the ER by subcellular fractionation using ELISA.	CHO cells	Overexpression
	[118]	Aβ_42_ production was detected by ELISA when APP was retained in the ER via an ER retention motif.	Human NT2N neuron	Overexpression
		Intracellular Aβ_42_ production detected by ELISA in the cells when APP is retained in the ER after BFA treatment (24 h) (note: no secretion)	Human NT2N neuron	Endogenous
	[119]	C99 (APPβ) before APP transport to the Golgi by immunoblotting after incubation at 15^0^C	Human NT2N neuron	Endogenous
		C99 (APPβ) detected in the ER fraction by immunoblotting when APP was retained via an ER retention motif.	Human NT2N neuron	Overexpression
	[120]	Aβ co-localised with an ER marker by IF.	N2a Mouse Neuroblastoma	Endogenous
	[121]	Aβ_40_ and Aβ_42_ were detected at the ER-mitochondria contact sites by subcellular fractionation.	C57BL6/J mouse brain	Endogenous
Golgi/TGN	[122]	Secretion of Aβ was blocked by Golgi perturbation using monensin and BFA.	COS-1 cells	Overexpression
	[115]	Aβ_40_ was detected predominantly at the TGN by electron microscopy using a specific antibody.	Primary rat hippocampal neurons	Endogenous and overexpression
	[116]	Aβ_40_ and Aβ_42_ co-localised with a Golgi marker by IF.	Primary rat cortical neurons	Endogenous
		Aβ_40_ and Aβ_42_ were detected in the Golgi (TGN) fraction by subcellular fractionation.	Primary rat cortical neurons	Endogenous
		Aβ_40_ and Aβ_42_ were detected in the Golgi fraction by subcellular fractionation.	N2a Mouse Neuroblastoma	Overexpression
	[117]	C99 (CTFβ) was increased in the Golgi-rich fraction by subcellular fractionation from PSEN1 KO.	Fibroblast from KO mouse embryo	Endogenous
		Aβ_40_ and Aβ_42_ were found in the Golgi fraction by subcellular fractionation using ELISA.	CHO cells	Overexpression
Cell surface	[115]	Aβ_40_ and Aβ_42_ were detected on cell surface by EM using two specific antibodies.	COS-7 cells	Overexpression
Endosomes and lysosome	[58]	Full-length APP and CTFs were detected in purified lysosomes.	Endothelial cell (HUVEC)	Endogenous
	[53]	Full-length APP and CTFs were identified in purified, fractionated clathrin-coated vesicles (CCV).	PC12 cells	Endogenous
	[123]	Aβ was detected in multivesicular bodies (MVB)/ late endosomes by EM using an Aβ antibody.	N2a Mouse Neuroblastoma	Endogenous
	[78]	Aβ and CTFs were detected in isolated exosomes.	Primary rat cortical neurons	Endogenous
	[124]	Aβ was detected in MVB in hippocampal and cortical neurons by EM	Mouse, rat and human brain tissue	Endogenous
	[39]	Full-length APP and CTFs were detected in early endosomes in the soma using STED imaging.	Primary mouse hippocampal neurons	Endogenous
		Aβ_42_ was detected in late endosome-lysosomes in the soma using STED imaging.		

Intracellular locations of β-secretase cleavage products of APP detected by subcellular fractionation, immunofluorescence and/or electron microscopy. The APP cleavage products detected are listed based on location, including ER, Golgi, cell surface and endosomes. Detection of C99 (CTFβ) and Aβ are shaded in yellow and red, respectively.

### β-Secretase processing of APP in the secretory pathway: ER and Golgi

Studies in 1990s first identified APP and the products from amyloidogenic processing pathway in the early secretory pathway, enriched in the ER and the Golgi apparatus, in a range of cell types including immortalised and primary neurons (Table 2). Aβ and C99 (CTFβ) were detected in the ER and/or Golgi network using subcellular fractionation, IF and/or EM (Table 2). In addition, and consistent with the possibility of APP processing in the secretory pathway, some studies have shown that the endocytic system is not necessary for BACE1-mediated APP cleavage to occur. A mutated BACE1, with defective BACE1 internalisation motif, was still able to cleave APP, indicating that the endocytosis of BACE1, *per se*, is not essential for the β-secretase cleavage of APP [98]. In addition, interrupting retromer-mediated retrograde transport of APP from the early endosomes back to the TGN resulted in an accumulation of APP in early endosomes and a diminution of β-secretase processing of APP, suggesting that the TGN represents an important hub for β-secretase cleavage [59]. The overexpression of BACE1 *in vivo* have also demonstrated that APP processing by BACE1 can occur in early secretory pathway in mouse neurons [125].

### β-Secretase processing of APP on the cell surface

While both BACE1 and APP can reach the PM, the processing of APP by the β-secretase on the cell surface has not been rigorously demonstrated, partly because the pH at the PM does not favour β-secretase activity. However, there is some evidence for Aβ_40_ and Aβ_42_ production on the cell surface in non-polarised COS cells [115]. Expression of a defective dynamin (dyn K44A) to inhibit APP endocytosis surprisingly resulted in an increased production of C99 (CTFβ) as well as Aβ secretion. Although this result may suggest that APP can be cleaved at the PM [126], dynamin deficiency does not abolish endocytosis completely (as some endocytosis pathways are unaffected) and the impact of the dynamin mutant on other aspects of APP trafficking, such as exit from the TGN, was not assessed. BACE1-APP interactions have been observed at the cell surface by FRET [127]. Hence, it is still possible that some β-cleavage can take place at the PM, albeit at low levels given the sub-optimum pH conditions for β-secretase catalytic activity. As the area of the PM is considerably larger in neurons compared with non-polarised cells, β-secretase processing of APP at the PM might be more relevant in neurons.

### β-Secretase processing of APP in the endosomal system

The early endosomes and the endosomal-lysosomal system are considered to be prime sites for BACE1 cleavage, especially given the acidic pH of 4–6 [105], which favours aspartyl proteases [106]. The role of the endo-lysosomal system in APP processing was proposed after the observation that the disruption of lysosomal function (with ammonia chloride and leupeptin treatments) limits the production of C-terminal APP fragments [128]; although, this finding has been challenged [122]. Notably, purified lysosomes were found to contain a range of APP cleavage products [58], and application of chloroquine, a lysosome inhibitor, resulted in accumulation of APP in lysosomes, suggesting a role of lysosome in clearing APP [129].

The endocytosis of APP has been extensively studied in various cell models and there is considerable evidence for dysfunctional transport in the endosome-lysosome pathway in AD (reviewed in [130,131]). Full-length APP and its CTFs have been identified in purified, fractionated clathrin-coated vesicles (CCV) [53]. Surface biotinylated APP can be internalised [58], and immunolabelling of cell-surface APP also demonstrated endocytosis and recycling of APP from the membrane [132]. BACE1-APP interaction has been observed in the early endosomes by FRET, supporting that the early endosomes are a site for the β-secretase cleavage of APP [127]. Moreover, *in vivo* inhibition of clathrin-mediated endocytosis reduced Aβ levels in the brain interstitial fluid of mice [133]. The increase of Aβ secretion in the brain interstitial fluid caused by induced synaptic activities can also be repressed by the inhibition of endocytosis [133]. In addition, spatial temporal analysis of the trafficking and processing of APP detected a low level of β-secretase processing of APP in the early endosomes [48]. Collectively, these results demonstrate that endosomes are a site for APP processing by the β-secretase.

APP and BACE1 cytoplasmic sorting motifs and the adaptors and pathways for endocytosis of APP and BACE1 have been intensively investigated (reviews by [43,45]). Early studies indicated that the endocytosis of APP in non-neuronal cell lines is mediated by a GYENPTY motif [134], an extension of the more classical NPXY motif which can mediate internalisation via interaction with the adaptor AP-2 [135]. However, a recent report has found that in primary rodent neurons the GYENPTY motif does not contribute to APP internalisation and suggested that a clathrin independent endocytic pathway for APP operates in primary neurons [93]. Notably, the GYENPTY motif does conform to the sequence motif of øxNxx[YF] recognised by sorting nexin 17 (SNX17) for recruitment by the Commander complex for recycling [136–138] either directly to the cell surface or possible indirectly via recycling endosomes or the TGN. There has also been some controversy whether BACE1 internalisation was mediated by the clathrin mediated endocytosis [97] or a clathrin/AP-2 independent ARF6 pathway [139]. A recent study [140] has shed some light on these disparate findings and demonstrated that AP-2 was not required for BACE1 endocytosis in neurons but was required for the subsequent trafficking of BACE1 to the lysosomes; indeed, in the absence of AP-2, endocytosed BACE1 was rapidly recycled to the cell surface [140]. Also in a recent study, Aow et al. [93] demonstrated that internalised APP from the somatodenritic cell surface of primary neurons may not represent the major source for production of Aβ [93]; this finding is compatible with the observation that newly synthesised APP is predominantly transported directly to early endosomes [47,48,129]), and which also highlight differences between non-neuronal and neuronal systems.

A number of late-onset AD risk factors have been identified which have a role in endosomal sorting. for example, BIN1, CD2AP, PICALM, SORL1, components of retromer and the retromer associate protein Rab 7A [4,35,141–143]. The sorting protein receptor, SORL1, is a risk factor associated with AD and functions in the endosomal sorting of APP and the regulation of APP processing [144–148]. The link between retromer, endosomal sorting and pathology of Alzheimer's has now been well established in several models, including in mouse models [149,150]. Of note, recent advances have shown that Bin1 and CD2AP control the transport and lysosomal degradation of APP in axons and dendrites, respectively [151]. Loss of function of Bin1 or CD2AP variants associated with AD results in the characteristic enlargement of early endosomes and increases in Aβ generation in both axons and dendrites [151]. Phospholipase D3 (PLD3) is another late onset disease risk factor, which is enriched in neuronal lysosomes. PDL3 deficiency results in lysosomal dysfunction and downstream perturbations in mitophagy, cholesterol metabolism and APP processing [152], demonstrating the importance of endosomal-lysosomal homeostasis for regulation of APP processing. In addition, a protein associated with clathrin-mediated endocytosis, PICALM, has been identified from a GWAS study as a factor with a protective role in AD [153].

### Location of α-secretase processing

In comparison with BACE1 processing of APP, the α-secretase cleavage of APP has been less studied, probably because it does not directly contribute to the progression of AD. However, the balance between the amyloidogenic and non-amyloidogenic pathways is important to appreciate the regulation of Aβ production. Knowledge of the intracellular sites of APP α-secretase cleavage is relevant to define the cell biology of non-amyloidogenic processing pathway and to explore the possibility of diverting APP processing from the amyloidogenic pathogenic pathway to the non-amyloidogenic pathway [154]. The disintegrins and metalloproteases ADAM10 and ADAM17 have been identified as the major α-secretases for APP [23,155], and these ADAM proteins are localised throughout a range of membrane-bound organelles, including the Golgi, PM and endosomes (reviewed in [156,157]).

### α-Secretase processing of APP on the cell surface

In early studies, PM localised APP was observed to be cleaved by membrane-bound proteases [51,158] and α-secretase processing of APP was conventionally described as occurring predominately at the PM. After a 20°C temperature block to retain APP in the TGN in neuroblastoma cells, the soluble α-secretase processing product sAPPα was not detected, suggesting that α-secretase cleavage of APP did not occur in the secretory pathway [159]. However, secretase activities have subsequently been shown to be severely compromised at 20°C [48,160]. Biotinylated inhibitor AMG110552, considered at the time to inhibit α-secretase only at the PM, was shown to abolish sAPPα production by ∼90% [159]. However, as biotin diffuses freely across the PM, the biotinylated AMG110552 inhibitor may also enter the cell. When APP internalisation was inhibited by (1) overexpression of a truncated APP without its cytoplasmic tail or (2) by the expression of a defective dynamin to inhibit endocytosis, there was an increased production of sAPPα [158] and C83 (CTFα) [126], respectively. It is important to note that the truncation of the APP cytoplasmic tail could result in the accumulation of APP in the TGN, and the defective dynamin may also compromise efficient export of cargo from the TGN, both concerns were not thoroughly assessed.

### α-Secretase processing of APP in the secretory pathway

A number of studies have shown that α-secretase processing of APP can occur at different intracellular sites in the secretory pathway in addition to the PM. Indeed, the α-secretase cleavage fragment sAPPα has been detected in intracellular compartments in polarised MDCK cells [161] and in the ER after APP was tagged with an ER retention motif [162].

More recently, the Golgi apparatus has been proposed as a new hub for APP α-secretase cleavage. The level of secreted sAPPα is increased in both cultured cell lines and primary mouse cortical neurons following an artificial retention of APP in the TGN, by knockdown of adaptor proteins, indicating that endogenous APP can be cleaved by the α-secretase in the TGN [163]. α-Secretase processing in the TGN was previously supported by the finding that an APP chimera containing the cytoplasmic tail of furin, to retain the chimeric protein in the TGN, released sAPPα [164]. In addition, α-secretase processing has been investigated following a block in post-Golgi transport. Firstly, inhibition of the exocytosis by the expression of mutants of the SNARE protein, syntaxin-1A, demonstrated that α-secretase processing can occur at normal rates without the need of the delivery of APP to the PM [165]. Secondly, a dominant-negative Rab6 mutant, which impedes post-Golgi transport, resulted in an increase of sAPPα [166]. Collectively, this evidence strongly implies that α-secretase processing of APP can take place in the early secretory pathway, especially in the Golgi/TGN in non-polarised cells. Further studies are required to determine the relative contribution of α-secretase cleavage of APP within intracellular compartments and at the cell surface. Application of imaging techniques to specifically detect soluble APPα in real time would be informative.

In summary, both α- and β-secretase cleavage of APP can take place at multiple cellular sites. The endosomal system is often considered as the predominant site for β-secretase processing under steady state conditions [167]. However, mutation of APP can modify the location of APP cleavage sites highlighting a role of the secretory pathway in the processing of APP which is discussed below.

## Role of membrane lipids in APP trafficking and processing

The role of lipids, especially cholesterol, has received considerable attention in promoting Aβ production. The brain is particularly enriched in cholesterol compared with other tissues [168] and high levels of cholesterol are associated with AD [169]. Some of the genetic risk factors associated with late onset AD, such as the *APOE4* allele, regulate cholesterol metabolism [170]. There is now considerable evidence that cholesterol levels are correlated with Aβ production. Membrane cholesterol plays a key role in the organisation of lipid domains and in the partitioning of membrane proteins within the membrane bilayer. Cholesterol, together with saturated lipids and sphingolipids, are major components of lipid rafts, microdomains containing highly ordered lipid aggregates which can laterally diffuse within a pool of disordered membrane lipids [171]. APP, BACE1, α-secretase and γ-secretase, as well as Aβ peptide, have been shown to preferential partition between the disordered and ordered membrane domains and, as processing requires direct access of secretases with their APP substrate, the relative distribution of enzyme and substrate is critical to the regulation of Aβ production [172,173]. Reducing cholesterol levels in a number of different cell lines, including mouse neuronal cell lines, resulted in inhibition of BACE1 cleavage and reduced Aβ levels [171,174,175] and in brains of transgenic mice [176], indicating that β-secretase cleavage of APP could occur within lipid rafts. In addition, a number of studies have shown that increased cholesterol levels promote APP and BACE1 co-localisation in lipid rafts (see review [170]). Cholesterol-rich lipid rafts play a key role in protein sorting events in the secretory and endocytic pathways [35,177]; and changes in the density of ordered lipid microdomains could result in enhanced clustering of APP and secretases in either the TGN or early endosomes. *APOE4* neurons differentiated from human induced pluripotent stem cells exhibit elevated Aβ secretion compared with isogenic *APOE3* neurons [178], indicating a potential role for cholesterol in human neurons in regulating the access of secretases to the APP substrate. A recent quantification of the affinity of C99 in different membrane domains in cell-derived giant PM vesicles has demonstrated that a majority of C99 partitioned into membrane disordered domains which highlights the need for additional studies to fully define the complex role of the lipid environment in AD [179].

Super-resolution microscopy using the Retention Using Selective Hooks (RUSH) system has demonstrated that newly synthesised APP and BACE1 are largely segregated on exit from the ER and throughout the Golgi [40], suggesting that APP and the β-secretase are partitioned into distinct lipid domains early in the secretory pathway. Palmitoylation of APP and the secretases has been reported to modify their enrichment in lipid domains, which adds a further level of regulation to amyloidogenic processing [180]. High levels of cholesterol may also promote a direct interaction of cholesterol with APP, influencing APP dimerisation and trafficking [181]. Collectively, these findings emphasise the importance of cholesterol in Aβ production. The impact of cholesterol levels in experiments using primary neurons would be worthwhile to be monitored.

## Impact of familial AD mutations on the location of APP processing

**Figure 4.**
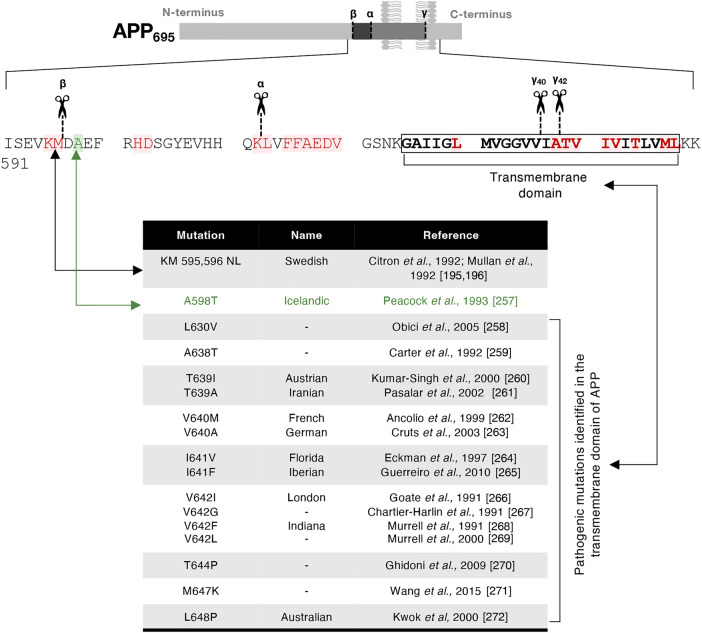
Mapping of mutations in APP. The protein sequence of the transmembrane domain (TMD, **bold**), and the flanking sequences of the cytoplasmic tail the luminal domain of APP_695_ are shown. The secretases cleavage sites are illustrated as in Figure 1. The Swedish mutation, a double mutation substituting K595 and M596 to N595 and L596, is mapped adjacent to the β-cleavage site. The protective Icelandic mutation, A598T, is illustrated in green. Other pathogenic APP mutations identified in familial Alzheimer's disease are labelled in red. Details of the pathogenic mutations which map in the transmembrane domain of APP are identified in the figure table.

A number of genes have been identified associated with early-onset familial AD, and which are inherited in an autosomal dominant manner. The majority of early onset disease-related mutations map to either the coding region of APP or γ-secretase (PSEN1, PSEN2), highlighting the importance of APP processing in AD initiation and progression [182] (Figure 4). Other mutations, which indirectly influence disease progression, have been identified in proteins regulating membrane trafficking or Aβ clearance (i.e.: PICALM, SORL1) (reviewed in [15,183]). APP disease-associated mutations can be used as powerful tools to investigate the pathogenesis of AD and the molecular and cellular mechanisms underlying the disease.

Many pathogenic mutations of APP (114 mutations to date) have been identified in a close proximity or within APP transmembrane domain [15] (Figure 4). Given the proximity of the mutations to the secretase cleavage sites in APP, many studies have focused on altered secretase substrate binding and/or catalytic activity of β- and/or γ-secretases caused by the mutations. For example, the pathogenic ‘Swedish’ mutation (APPswe) (discussed in more detail in the next section) is associated with an increased cleavage by BACE1 and the pathogenic ‘London’ mutation (V642I) has been shown to display altered β- and γ-cleavage with increased levels of sAPPβ and Aβ [184]. The London mutation results in weaker binding of the APP-PSEN1 interaction as analysed by computer-based simulation [185]. The alteration of an interaction between APP and PSEN1 has also been demonstrated for other APP mutations within the transmembrane domain close to the γ-secretase processing sites by cryoEM [186], and is associated with increased production of C83/C99 (CTFs) and an increased Aβ_42_/Aβ_40_ ratio [187]. Pathogenic PSEN mutations destabilise the γ-secretase-APP complex during sequential γ-secretase processing and, by enhancing the dissociation of PSEN with APP, the longer toxic Aβ42 peptide is favoured compared with the non-mutated PSEN1 (wild-type) [188].

Additional mechanisms have been proposed for other pathogenic APP mutations. For example, V640A (German) and V640M (French) mutations change the conformation of the dimer of the APP transmembrane domain and favour the production of Aβ42, as shown by NMR [189]. In addition, the aggregation potency of Aβ is influenced by mutations within the Aβ domain, i.e.: the A617G (Flemish) mutation [190,191] or D603N/H602R mutation [192,193]. The profile of Aβ peptides have been shown to differ for some of the familial APP mutations, indicating that different Aβ species are produced from APP carrying different familial AD mutations [194]. A detailed list of publications on each mutation is summarised in the online data base ‘Alzforum’ (https://www.alzforum.org/mutations/app) [15]. Findings on APP Swedish mutation are reviewed in detail below, as it is the most intensively studied of the familial APP mutations.

### The pathogenic familial APP Swedish mutation

The Swedish mutation (APPswe) was identified from two Swedish families diagnosed with early-onset AD [195,196]. It is a double mutation, substituting K595 and M596 to N595 and L596 in the APP_695_ isoform, at a location adjacent to the β-secretase cleavage site [195,196] (Figure 4). The Swedish mutation is pathogenic and is associated with elevated secretion of Aβ and neuronal abnormality [197], however the underlying cellular mechanism behind the effect is only partially understood.

### β-Secretase proteolytic activity is enhanced by the APP Swedish mutation

Studies during the 1990s showed that the APP Swedish mutation resulted in an increased level of Aβ in both non-neuronal cells and a human neuroglioma cell line [195,198–200]. Given the proximity between the position of the double mutation and the β-secretase cleavage site in APP (Figure 4), a plausible hypothesis for the increased Aβ production from APPswe is that the mutation amplified BACE1 enzymatic cleavage. Increased catalysis of APPswe by BACE1 was demonstrated from studies using purified BACE1; purified BACE1 tagged with an IgG C-terminal tail was found to cleave APPswe at a faster rate than the wild-type APP (APPwt) [24]. Using synthetic peptides as substrates, the rate of BACE1 cleavage was 10-fold higher for the APPswe peptide than for the equivalent APPwt peptide [201,202]. The catalytic efficiency of BACE1 (*k*_cat_/*K_m_*) is also significantly higher (60-fold) for APPswe than APPwt [203].

3D modelling of the BACE1 structure with APP as a substrate showed that hydrophobic residues in the protease domain of BACE1 may form a pocket to bind the hydrophobic leucine residue of APPswe (KMàNL) [204,205]. A common view has been that increased proteolytic activity may be solely responsible for the pathology of APPswe. However, within the context of the cell, pathogenesis induced by mutations can be multifaceted.

### Trafficking and processing of APPswe in the secretory pathway

In addition to the changes in BACE1 proteolytic activity, changes in the intracellular localisation of APPswe have been investigated in non-neuronal cell lines, mouse neuroblastoma cells as well as and human neuroglioma cells. A recent and important observation confirmed that APPswe is predominantly cleaved in the secretory pathway whereas APPwt is preferentially cleaved in the endocytic pathway [48]. Preferential cleavage of APPswe in the secretory pathway was initially suggested by the detection of β-secretase cleavage products of APPswe [160,206,207]. In cells expressing APPswe, metabolically labelled Aβ was detected in the secretory pathway [206]. In addition, APPswe-derived Aβ was enriched in the fractions containing Golgi/TGN and nascent post-TGN vesicles, suggesting that the Golgi might be the major location for the processing of APPswe and the generation of Aβ [160]. An antibody specific to sAPPswe-β (secreted sAPPβ derived from APPswe) detected sAPPswe-β in Golgi-derived vesicles. Also, sAPPswe-β did not co-localise with internalised transferrin, indicating that β-secretase cleavage of APPswe takes place in the secretory pathway rather than the endocytic pathway [207]. In addition, cells expressing a truncated APPswe without a cytoplasmic domain (which cannot be endocytosed) secreted more sAPPswe-β than full length APPswe, suggesting that endocytosis of APPswe was not necessary for sAPPswe-β production [208].

The involvement of the secretory pathway directly in the processing of APPswe has also been demonstrated using different experimental approaches. In a pulse-chase experiment, labelled C99 (CTF-β) derived from APPswe appeared much earlier than C99 derived from APPwt in the chase, and possibly within the *medial*-Golgi [209]. Using the RUSH system to monitor the anterograde trafficking of newly synthesised APPswe in real-time, APPswe has been shown to transit through the Golgi less efficiently than APPwt and is associated with enhanced amyloidogenic processing [48]. Interestingly, the inhibition of BACE1 restored APPswe anterograde trafficking profile to that of APPwt [48]. This study revealed preferential intracellular locations for the cleavage of APPwt and APPswe and highlighted the Golgi apparatus as a major processing site for APPswe. In addition, the interaction of APPswe and BACE1 in the Golgi apparatus has been demonstrated by FRET [127]. In cells co-expressing APPswe and a familial PSEN1 mutation, Aβ_42_ was enriched in the TGN-transport vesicles fractions (Rab8 positive) [210] whereas only a low level of Aβ_42_ derived from APPswe was detected in the early endosome fraction (Rab5 positive), confirming that APPswe-derived Aβ_42_ was produced preferentially in the secretory pathway rather than in the endocytic pathway [210].

Abnormalities in the secretory organelles are observed concomitant with the pathology associated with the familial APPswe mutation. The Golgi apparatus is fragmented in hippocampal tissues of mice expressing both APPswe and the familial PSEN1 mutation, PSEN1Δ9 [211]. Overexpression of APPswe induces an up-regulation of ER-stress response [212]. These finding indicate that the phenotype of APPswe could be linked to ER/Golgi abnormalities.

### Trafficking and processing of APPswe in the endocytic pathway

A role for the endocytic pathway in the processing of APPswe has also been suggested. Surface radioiodination of cells expressing APPswe suggested that endocytosed APPswe can contribute to the production of Aβ [206]. The late endocytic pathway is altered in primary mouse cortical neurons expressing APPswe, but not APPwt [110]. In addition, Aβ produced by overexpressing APPswe has been found to be associated with isolated exosome preparations [78,123] and exosomes are commonly derived from multivesicular bodies (MVB) or late endosomes, suggesting a pathway for Aβ produced in the endocytic pathway to be secreted. Although there is some evidence that the endocytic pathway may contribute to the processing of APPswe, there is compelling evidence that the secretory pathway plays a major role in the amyloidogenic processing of APPswe. It is also worth noting that the secretion of Aβ from the Golgi apparatus via constitutive transport is likely to be very efficient, whereas the pathways for secretion of Aβ generated within the endosomal system are likely to be very inefficient as the luminal content is delivered to the late endosomes/lysosomes.

Based on the above findings, it is important that the analysis of the trafficking and processing of APPswe is not generalised to APPwt. In some studies, APPswe has been used as a ‘boost’ system for enhanced Aβ production with conclusions generalised to APPwt, under the assumption that the intracellular trafficking and location of cleavage are the same for APPwt and APPswe. There is now substantial evidence to indicate that APPwt and APPswe differ in their major intracellular sites of BACE1 processing [48,213].

### Trafficking and processing of APP in healthy and patient induced pluripotent stem cell-derived neurons

Recently, human induced pluripotent stem cell (iPSC)-derived neurons have emerged as a powerful human neuronal model to investigate neurodegenerative diseases, as it allows characterisation of patient iPSC-derived neurons and comparison with healthy iPSC-derived neurons. Using a proximity ligation assay, interaction of APP and BACE1 was observed in neural precursor cells as well as in healthy human iPSCs-derived neurons [214]. The distribution of APP in the soma and axons has been analysed [215], and APP was detected in Rab11-positive recycling endosomes, which is proposed to mediate soma to axon transcytosis in neurons [215]. In healthy human iPSC-derived neurons, exogenous Aβ enhanced APP and BACE1 co-localisation in Rab11-positive recycling endosomes, visualised by APP-VN and BACE1-VC interactions [216], and indicating that Aβ can alter the localisation of APP in human neurons.

A number of iPSCs derived from patients carrying familial APP mutations have been differentiated into neurons and characterised *in vitro*. Relevant observations from these studies include: (1) *Altered APP processing.* In patient iPSC-derived neurons carrying the APP London mutation (V642I in APP_695_, V717I in APP_770_), both β- and γ-secretase cleavage of APP are elevated compared with controls [184]. Human iPSC-derived neurons carrying the familial AD *PSEN1* (γ-secretase) mutations or a knock-in of *APPswe* displayed increased Aβ and Tau production [217]. (2) *Altered APP localisation.* In human iPSC-derived neurons carrying a PSEN1 familial mutation, APP was concentrated in the soma and decreased in axons [215]; endocytosis and transcytosis of APP from soma to axons was also impaired [215]. (3) *Organelle abnormalities*. Enlarged early endosomes have been observed in neurons from post-mortem brain tissues of Alzheimer's patients [218] and from human iPSC-derived neurons from both sporadic and familial AD [219]. Enlarged MVBs have also been detected in primary mouse neurons from an AD model of transgenic mice expressing two familial mutations (APPswe/PSEN1Δ9) [220]. Endosome enlargement and dysfunction were also observed in human iPSCs-derived neurons where the AD risk gene SORL1 was knocked out, indicating that the loss of the sorting receptor SORL1 influences AD pathogenesis [148]. In addition, Golgi abnormalities, especially Golgi fragmentation, is one of the earliest disease phenotypes displayed by human iPSC-derived neurons carrying familial AD PSEN1 mutations or a knock-in of *APPswe* [217]. (4) *Synaptic alternation*. There are conflicting reports on the effect of familial APP mutations on synaptic densities in neurons. In human embryonic stem cell derived neurons with knock-in of APPswe, the synaptic density was observed to increase compared with neurons expressing APPwt [197], whereas in another study in human iPSC-derived neurons with a knock-in of *APPswe*, synaptic density of neurons was reduced [217].

One important gap in knowledge is the precise impact of the familial *APP* mutants on trafficking and processing of APP. Many of the familial APP mutations are located within the transmembrane domain, which could alter their trafficking and affect the physiological function of APP. Notably, transmembrane domains can play a key role in cargo sorting, mediated by interactions with lipid subdomains [221,222]. Further studies are needed to fully appreciate the impact of familial *APP* mutants on the amyloidogenic pathway and determine the underlying mechanisms which contribute to enhance APP processing and Aβ production.

### Effect of presenilin mutations on APP trafficking and processing

The γ-secretase is a multi-subunit aspartyl protease, comprised of one catalytic core, presenilin 1 (PSEN1) or presenilin 2 (PSEN2), and three accessory subunits [223]. The core subunit, presenilin, has been detected in the early secretory pathway, namely in the ER [224], the ERGIC [225,226], and the Golgi apparatus [227]. There is considerable evidence that the functional γ-secretase four-subunit complex is assembled following ER exit [223,228,229]. *In vivo* and *in vitro* COPII budding assays demonstrated that dimeric γ-secretase subcomplexes were efficiently recruited into COPII vesicles for ER exit [230] and the dimers subsequently assembly in a post-ER compartment, probably the ERGIC. However, under certain conditions the γ-secretase may be able to function in the ER. Expression of the γ-secretase subunit, nicastrin, with an ER-retention signal was able to generate AICD, the γ-secretase product of an ER-retained APP [231], indicating that the ordered pathway for assembly of γ-secretase is important for spatial regulation of its activity. γ-Secretase activity has been reported in the ER-mitochondrial membrane contact sites [232], however, the basis for the location of active γ-secretase complex at this site, given the assembly pathway discussed above, is unknown.

Over 360 and 90 pathogenic mutations have been identified in *PSEN1* and *PSEN2*, respectively [15]. A detailed list of presenilin mutations and their pathology can be found in the ‘Alzforum’ data base [15]; most of the pathogenic *PSEN1* and *PSEN2* mutations are associated with an increased Aβ42 production *in vitro*. Many studies have focused on the effect of presenilin mutations on the identity of the neurotoxic Aβ species generated [233]. Also, presenilin 1, as an independent holoprotein, has many functions in addition to γ-secretase activity and disruption of any of these functions arising from the pathogenic mutations could contribute to AD [234,235]. Relevant to this review, there are a number of studies which have explored the effect of presenilin mutations on APP trafficking and/or location of APP γ-secretase processing.

Presenilin mutations have been shown to affect APP trafficking in the secretory pathway. In N2a mouse neuroblastoma cells expressing APPswe with either WT PSEN1 or PSEN1 deletion (*PSEN*−/−), trafficking of APPswe was altered by *PSEN1* deletion, indicating roles for γ-secretase in addition to processing [236]. In N2a cells expressing APPswe with either WT PSEN1 or the familial disease PSEN1 Δ9 mutation, which results in the exclusion of exon 9, the majority of Aβ42 was colocalised to Rab8-marked Golgi-derived vesicles in the late secretory pathway [210]. The artificial introduction of D257A or D385A mutations in the transmembrane domains of PSEN1, results in an inactive γ-secretase [237] and alteration of APP trafficking [238]. In addition, the familial AD PSEN1 A246E or M146V mutations can led to reduced level of APP delivered to distal neurites in primary mouse cortical neurons [236,239]. Knock-in of PSEN1 M167V in mouse neurons resulted in reduced levels of APP trafficking from the TGN compared with control mouse neurons [240]. Presenilin 1 mutations have been shown to also affect the trafficking and distribution of other AD-related neuronal proteins or substrates, including N-cadherin [241], TREM2 [242], and the APOE receptor apoER2 [243]. Increased expression of BACE1 was observed in *PSEN1* mutant knock-in mice and cultured cells [244,245] as well as changes in the distribution of mature BACE1 in the secretory pathway [244].

A basis for the existence of the two homologous PSEN1- and PSEN2-γ-secretase complexes has recently been revealed by the discovery that they have different intracellular locations; PSEN1 secretase is widely distributed throughout the cell whereas PSEN2 secretase is predominantly restricted to late endosomes and lysosomes [246]. The location of PSEN2 is mediated a phosphorylated acidic dileucine motif of PSEN2 which interacts with AP1 to mediate sorting from the TGN to late endosomes [246]. Notably some of the familial PSEN2 mutations increase the production of the more toxic longer Aβ42 species [246], and moreover, some of the familial PSEN1 mutation shifts its location to late endosomes and lysosomes and also increase production of Aβ42 [233,246].

PSEN1 mutations have also been reported to induce organelle and trafficking abnormalities in neurons. In patient iPSC-derived neurons with PSEN1 L150P or A79V mutations, Golgi fragmentation was observed as an early phenotype [217]. A range of PSEN1 familial mutation knock-ins (including M146V and A246E) are associated with enlarged endosomes in human iPSC-derived neurons [247]. Vesicular traffic was affected by the PSEN1 M146V mutation in mouse astrocytes [248], and mouse neurons which expressed the PSEN1 M146L mutation were more prone to ER and Golgi stress [249]. Overall, these findings indicate that familial AD PSEN mutations can affect the cell biology of APP processing, however, the molecular and cellular basis for the phenotypes of only some of these PSEN mutations have currently been defined.

## Summary and future directions

**Figure 5.**
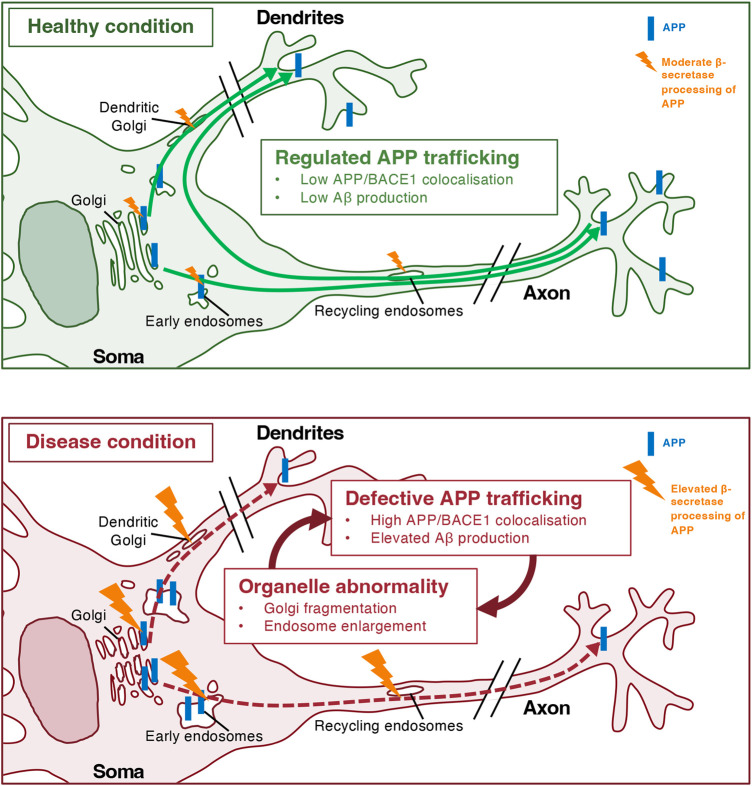
Model of the trafficking and processing of APP under healthy and disease conditions. Under physiological conditions, APP and BACE1 are well segregated in neurons. The partitioning of APP and BACE1 regulates APP processing and is associated with only low levels of Aβ production. Under conditions which promote Alzheimer's disease, dysregulation of APP trafficking and/or processing leads to elevated levels of APP and BACE1 convergence, resulting in an increased production of intracellular Aβ probably in the Golgi apparatus (somatic and dendritic), the early endosomes and in the recycling endosomes. The production of intracellular Aβ subsequently results in organelle abnormalities, including but not limited to Golgi disruption, endosome enlargement and lysosomal abnormalities. Perturbation of the architecture and membrane subdomains of the Golgi and endosomes may then lead to dysregulated protein sorting and trafficking, a reduction in the partitioning of APP and BACE1 in membranes and a further increase APP processing and production of Aβ. The intracellular stress caused by organelle abnormalities and Aβ production could have broader effects on the intracellular organisation and trafficking (i.e.: of synaptic receptors) which then lead to neuronal defects.

In summary, the secretase processing of APP can take place at multiple subcellular compartments. The Golgi apparatus and the endosomes are especially relevant for the amyloidogenic processing of APP. While APP is widely distributed in neurons in both axonal and somatodendritic domains, including synaptic membranes and vesicles, APP is particularly enriched in the Golgi and endosomes in the neuronal cell body (soma). Anterograde axonal transport and transcytosis deliver APP to the axons. APP trafficking and cleavage are mutually dependent events. Altered trafficking and processing of APP can increase the production of Aβ, affect the normal physiological function of APP and induce organellar abnormalities (Figure 5).

There are still many unanswered questions regarding the localisation and processing of APP in neurons and the Aβ secretion, some listed in Table 3. In non-polarised cell models, APP and BACE1 follow distinct post-Golgi trafficking pathways which tightly regulate the convergence APP and BACE1 and regulate Aβ production. Only limited quantitative studies have been carried out in neurons to explore when and where APP and BACE1 converge under physiological or disease conditions. Studies on APP transport and secretase cleavage in neurons have to date centred on the synthesis of APP in the soma, and the sorting events at the somatic, perinuclear Golgi and the axon initial segment. A key question is the potential contribution of a newly discovered ‘local’ dendritic secretory pathways on APP trafficking and processing. The ‘local’ dendritic secretory pathway consisting of dendritic ER and Golgi outpost structures has been identified in both rodent [250,251] and human iPSC-derived neurons [252], and have been proposed to play a role in rapid protein supply at remote locations and may play a critical role in regulating synaptic activity (reviewed in [253]). However, the contribution of this newly identified pathway to the trafficking of APP and the secretases remains still unclear.

**Table 3.** Unanswered questions relating to the intracellular trafficking and processing of APP

Questions
When and where do APP and BACE1 converge in primary neurons under physiological conditions and disease conditions? Is the intracellular distribution and processing location(s) of APP altered by disease-causing Alzheimer's disease mutations in primary neurons? Do APP mutations in the transmembrane domain affect cargo sorting in the Golgi? What is the role of cholesterol-rich membrane microdomains in regulating the segregation of APP and BACE1 of primary neurons? Is the alteration in the processing location(s) of γ-secretase a common consequence of PSEN mutations? Are there distinct pathways for the secretion of Aβ from the secretory and endocytic systems and what are their relative contributions to the extracellular pool? What are the underlying mechanisms by which elevated levels of intracellular Aβ modify the integrity and organisation of organelles in primary neurons? What is the impact of the somatic Golgi perturbation on the formation of Golgi outposts, anterograde protein transport, recycling, autophagy and synaptic activity?

Many pathogenic familial APP mutations are within the transmembrane domain. The effect of some mutations on the catalysis by the secretases have been studied, however, the effect of many of the mutations on the spatial-temporal regulation of APP trafficking and location(s) of APP processing is lacking, especially in human neurons. It is not known if APP mutations in the transmembrane domain affect the sorting of APP in the Golgi and/or in other compartments. The transmembrane domain of APP can potentially interact with various lipid domains of the membrane; for instance, C99 (CTF-β) has been proposed to partition in the disordered membrane phase [179] and can regulate cholesterol trafficking [254]. BACE1 and APPwt are segregated during their transport through the Golgi [40], which could be mediated by protein-lipid interactions. The role of membrane lipids in the segregation of APP and BACE1 and the impact of APP familial mutations on this segregation clearly requires further exploration.

Further investigation of the spatial-temporal interactions between APP and BACE1 in neuronal compartments is important to reveal the location(s) and pathway(s) for Aβ production and secretion, which remain poorly defined in neurons. In addition, it is not clear whether the intracellular site of Aβ production could influence the accumulation of toxic Aβ within the cell. Dysregulated production of Aβ may lead to organellar abnormalities, in particular in the endosomes and the Golgi apparatus [211,255]. Indeed, Golgi fragmentation is one of the earliest disease phenotypes observed in Alzheimer's patient iPSC-derived neurons [217]. The impact of Golgi fragmentation on the function of Golgi outposts, anterograde protein transport and synaptic activity needs to be investigated further. Cryo-EM tomography of human iPSC-derived neurons would provide a powerful approach to identify the effect of enhanced Aβ production on the structural integrity and organisation of organelles in neurons. Spatial-temporal analyses of the accumulation of APP products in defined organelles by proteomics will enhance our knowledge of processing events [256]. Moreover, a system biology approach, incorporating not only organelle proteomics, but also phosphoproteomics, lipidomics. RNA-seq analysis and cryo-EM tomography, would be a powerful approach to establish the link and timing between the perturbations of the different intracellular organelles.

In conclusion, understanding the spatial-temporal regulation of APP trafficking and the locations of APP processing in primary neurons is fundamental to uncover the mechanisms that are responsible for the initiation and progression of AD. Unravelling the membrane dynamics associated with the segregation, and convergence, of APP and BACE1 will also enhance the appreciation of the regulation of neuronal protein trafficking in neurobiology in general.

## Data Availability

As a review article, data sharing is not applicable to this paper.

## Open Access

Open access for this article was enabled by the participation of University of Melbourne in an all-inclusive *Read & Publish* agreement with Portland Press and the Biochemical Society under a transformative agreement with CAUL.

## Abbreviations

Aβ: amyloid-β
AD: Alzheimer's disease
AICD: APP intracellular domain
APP: amyloid precursor protein
APPwt: wild type APP
APPswe: Swedish APP mutation
BACE1: β-site APP-cleaving enzyme
CTFs: C-terminal fragments
EM: electron microscopy
ER: endoplasmic reticulum
IF: immunofluorescence
iPSC: induced pluripotent stem cell
MVB: multivesicular bodies
NPYss: neuropeptide-Y signal-sequence
NTF: N-terminal fragment
PM: plasma membrane
PSEN: presenilin
sAPP: soluble APP ectodomain
TGN: *trans*-Golgi network
